# Baseline Predictors of Crestal Bone Maintenance and Implant Stability Following Immediate Implant Placement With Platelet-Rich Fibrin: A Retrospective Observational Study

**DOI:** 10.7759/cureus.116926

**Published:** 2026-09-25

**Authors:** Uma Rodda, Seshu K Karri, Jhansi R Lotavath, Kandikatla C Shobhita, Viswanath Mylapoori, Korrapati Kishore

**Affiliations:** 1 Department of Oral and Maxillofacial Surgery, Government Dental College and Hospital, Kadapa, IND; 2 Department of Dentistry, Employees State Insurance Corporation (ESIC) Medical College and Post Graduate Institute of Medical Sciences and Research (PGIMSR), Chennai, IND; 3 Department of Oral Pathology, Government Dental College and Hospital, Kadapa, IND; 4 Department of Dentistry, Community Health Centre, Yarlagadda Ramavaram, IND

**Keywords:** bone density, crestal bone loss, dental implants, immediate implant placement, implant stability quotient, insertion torque, platelet-rich fibrin

## Abstract

Introduction

Immediate implant placement following tooth extraction is a predictable treatment approach, although peri-implant crestal bone remodeling and implant stability may vary according to local and patient-related factors. Platelet-rich fibrin (PRF) has been used as an autologous adjunct during immediate implant placement because of its potential to support bone and soft tissue healing. However, limited evidence is available regarding the baseline factors associated with crestal bone changes in immediate PRF-treated implants. This study aimed to evaluate crestal bone changes and implant stability, and to identify baseline factors associated with crestal bone changes following immediate implant placement with adjunctive PRF.

Materials and methods

This retrospective observational study included 52 single-unit implants placed immediately after tooth extraction and treated with adjunctive PRF. Clinical and radiographic records obtained at three and six months after implant placement were retrospectively evaluated. Crestal bone levels were assessed radiographically using the implant shoulder as a reference point. Implant stability was assessed using resonance frequency analysis and expressed as the Implant Stability Quotient (ISQ). Baseline bone density was recorded in Hounsfield units (HU), and the final insertion torque was recorded in Ncm. Changes in crestal bone levels and ISQ were assessed using paired-sample analysis. Multiple linear regression was performed to identify independent baseline predictors of total crestal bone change from baseline to six months.

Results

The mean age of the study population was 48.6 ± 11.3 years, comprising 29 (55.8%) males and 23 (44.2%) females. The mean bone density was 612.4 ± 118.7 HU, mean final insertion torque was 34.8 ± 6.9 Ncm, and mean baseline ISQ was 68.4 ± 4.2. Crestal bone loss was 0.18 ± 0.09 mm from baseline to three months, 0.14 ± 0.08 mm from three to six months, and 0.32 ± 0.13 mm from baseline to 6 months (all p < 0.001). The mean ISQ increased from 68.4 ± 4.2 at implant placement to 74.1 ± 3.8 at six months (mean ΔISQ = 5.7, 95% CI: 4.8-6.6; p < 0.001). Multiple linear regression demonstrated that a higher baseline ISQ (β = -0.011, p = 0.006), higher final insertion torque (β = -0.006, p = 0.015), and greater bone density (β =-0.0003, p = 0.022) were independently associated with lower crestal bone loss at six months post-loading. Age (p = 0.240) and sex (p = 0.653) were not significant predictors. The regression model explained 44.6% of the variance in total crestal bone change (R² = 0.446; p < 0.001).

Conclusion

In this retrospective cohort study, higher baseline implant stability, greater final insertion torque, and greater recipient site bone density were independently associated with lower crestal bone loss over six months following immediate implant placement with adjunctive PRF. These findings suggest that baseline mechanical and radiographic characteristics may be important factors in peri-implant crestal bone maintenance. Further prospective studies with larger sample sizes and longer follow-up periods are required to validate these associations.

## Introduction

Immediate implant placement following tooth extraction has become an increasingly accepted treatment approach in contemporary implant dentistry because it can reduce the overall treatment duration, minimize the number of surgical procedures, and help preserve natural alveolar architecture [1]. In addition to improving patient comfort and satisfaction, immediate implant placement may facilitate favorable esthetic outcomes, particularly in the anterior maxillary region, where the preservation of hard and soft tissue contour is critical [2]. Despite these advantages, successful osseointegration and long-term implant survival depend on the adequate preservation of the peri-implant bone and favorable soft tissue healing during the early postoperative period [3]. Physiologic remodeling following tooth extraction may result in dimensional changes in the alveolar ridge and peri-implant crestal bone, which can influence implant stability and esthetic outcomes [4].

Platelet-rich fibrin (PRF) is a second-generation autologous platelet concentrate that has gained considerable attention as a biologically active adjunct in regenerative dentistry [5]. Prepared without anticoagulants or biochemical additives, PRF forms a fibrin matrix enriched with platelets, leukocytes, cytokines, and multiple growth factors, including platelet-derived growth factor, transforming growth factor-β, and vascular endothelial growth factor [6]. These bioactive components are gradually released from the fibrin matrix and may promote angiogenesis, fibroblast proliferation, collagen synthesis, and osteoblast activity. Consequently, PRF has been investigated as an adjunct to implant therapy because of its potential to enhance soft tissue healing, support bone regeneration, reduce postoperative discomfort, and improve the biological environment surrounding dental implants [7].

Previous studies have reported favorable outcomes with the use of PRF during immediate implant placement, including improved soft tissue healing, enhanced early implant stability, reduced postoperative discomfort, and preservation of the peri-implant crestal bone [8,9]. However, the available evidence remains heterogeneous because of differences in study populations, PRF preparation protocols, implant systems, follow-up periods, and outcome-assessment methods [10]. Although the clinical outcomes of PRF-assisted immediate implant placement have been investigated, less attention has been directed toward identifying baseline clinical and radiographic characteristics that may be associated with subsequent crestal bone changes. Factors such as primary implant stability, insertion torque, and local bone density may influence the early biological and mechanical behavior of an implant and may therefore contribute to variations in peri-implant crestal bone remodeling.

Therefore, the present retrospective observational cohort study was conducted to evaluate crestal bone changes and implant stability over a six-month period following immediate implant placement with adjunctive PRF. The primary objective was to assess the change in crestal bone level from implant placement to six months. The secondary objectives were to evaluate changes in crestal bone levels between baseline and three months and between three and six months, assess the change in implant stability quotient (ISQ) over six months, and determine whether baseline ISQ, final insertion torque, recipient site bone density, age, and sex were independently associated with six-month crestal bone changes.

## Materials and methods

Study design and setting

This retrospective observational cohort study was conducted at the Department of Oral and Maxillofacial Surgery, Government Dental College and Hospital, Hyderabad, Telangana, India. The archived clinical and radiographic records of patients who underwent immediate implant placement following the extraction of a single tooth with adjunctive PRF between January 2015 and October 2017 were reviewed for this study. The study included eligible cases treated during the predefined study period for which complete baseline, three-month, and six-month clinical and radiographic records were available. All implant procedures were performed according to a standardized clinical protocol by an experienced oral and maxillofacial surgeon. This study was conducted in accordance with the ethical principles of the Declaration of Helsinki, and ethical approval was obtained from the Institutional Ethics Committee (GDCH-IEC/PG/1715, dated November 25, 2017). Individual informed consent was formally waived by the Institutional Ethics Committee because this study involved retrospective review of existing clinical records without any additional intervention or participant contact. PRF application was performed as part of routine clinical care and not specifically for the present investigation. This study was conducted through a retrospective review of routinely collected clinical and radiographic records.

Study population and eligibility criteria

Patients aged ≥18 years who underwent extraction of a single maxillary or mandibular tooth, followed by immediate implant placement, were included in the study. Eligible cases had adequate alveolar bone volume to permit immediate implant placement and achieve primary stability without the need for simultaneous guided bone regeneration or other bone augmentation procedures. Only cases treated with adjunctive PRF at the time of immediate implant placement and with complete clinical records, including insertion torque and implant stability measurements, together with standardized radiographic records at baseline, three months, and six months were included. Recipient sites classified as type II or type III on preoperative cone-beam computed tomography (CBCT) assessments (Carestream Dental India Pvt Ltd, Mumbai, India) were eligible.

Patients with type I or IV bone, simultaneous bone grafting, guided bone regeneration, sinus augmentation, or other augmentation procedures were excluded. Patients with uncontrolled diabetes mellitus, smoking exceeding 10 cigarettes/day, a history of periodontitis, active periodontal or peri-implant infection, previous radiotherapy involving the jaws, or systemic or local conditions likely to interfere substantially with wound healing, osseointegration, or bone remodeling were excluded. Patients with incomplete clinical or radiographic records, inadequate image quality for reliable measurements, failure to complete the six-month follow-up, or implant loss before the scheduled assessment were also excluded from the study.

Participant screening and exclusion

A total of 122 PRF-treated immediate implant cases were screened during the archival study period. After applying the predefined eligibility criteria, 52 patients with single-implant placements were included in the final analysis. The remaining 70 cases were excluded because of multiple implants placed in the same patient (n = 32), incomplete or missing clinical records or radiographs at one or more scheduled follow-up visits (n = 17), failure to complete the six-month follow-up assessment (n = 18), or immediate implant loss or failure before the six-month evaluation (n = 3). The participant screening, eligibility assessment, and exclusion process are summarized in Figure 1.

**Figure 1:**
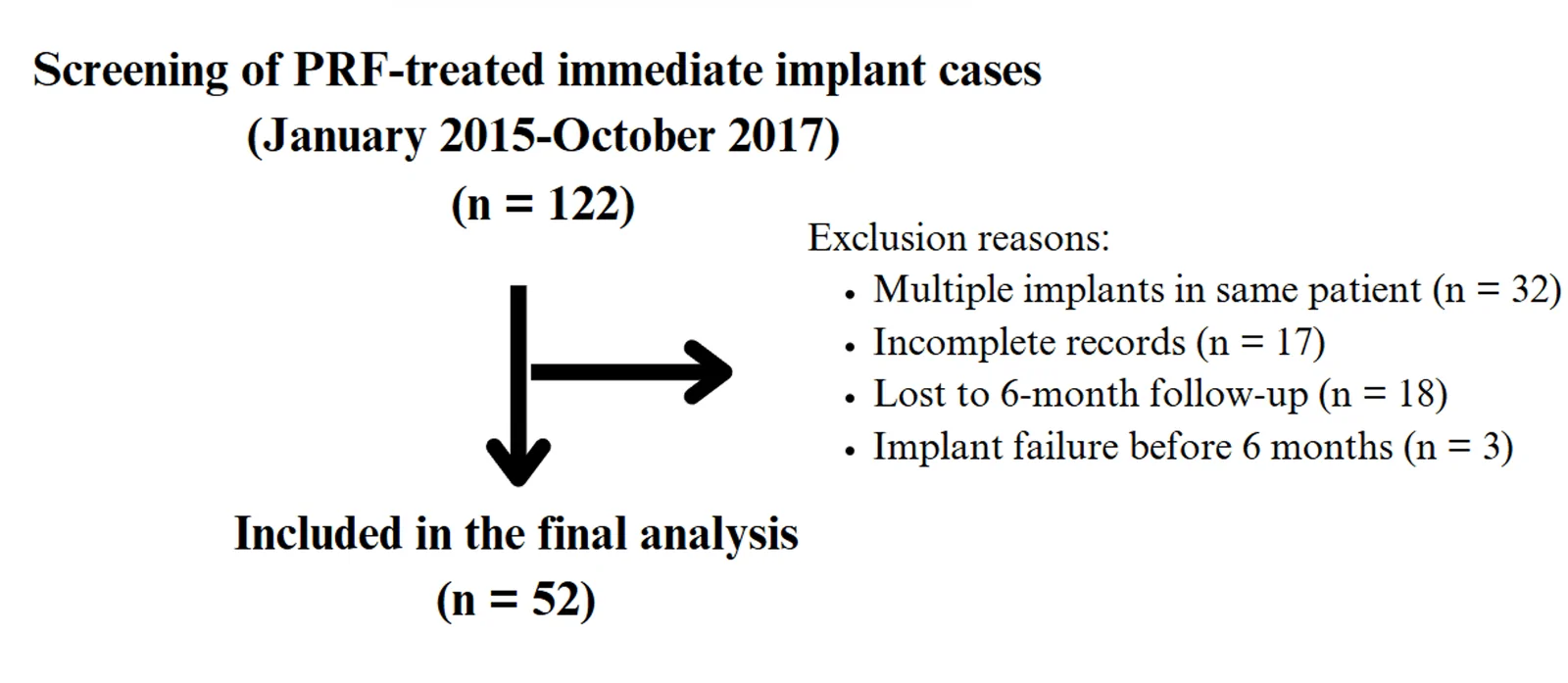
Screening of PRF-treated single implant cases Image created with Canva (Canva, Sydney, Australia). PRF - platelet-rich fibrin

Preoperative assessment

Detailed medical and dental histories were reviewed from the archived clinical records, followed by evaluation of preoperative radiographs and CBCT images. Preoperative investigations included intraoral periapical radiographs, orthopantomography, and routine hematological investigations. CBCT was used to evaluate the available alveolar bone dimensions, anatomical limitations, recipient site bone density, and suitability for immediate implant placement. The implant diameter and length were selected according to the available bone volume, socket dimensions, and prosthetic requirements.

Immediate implant surgical protocol

Surgical procedures were performed under strict aseptic conditions. Local anesthesia was achieved using 2% lignocaine hydrochloride with 1:80,000 adrenaline (Lignox®, Warren Pharmaceuticals Pvt. Ltd., Vadodara, Gujarat, India). Atraumatic tooth extraction was performed using periotomes and luxators to preserve the integrity of the buccal and lingual cortical plates. Following extraction, the socket was thoroughly debrided and irrigated with sterile normal saline, and the integrity of the socket wall was clinically confirmed. The dimensions of the extraction socket were assessed clinically and correlated with preoperative radiographic findings to determine appropriate implant dimensions.

Implant placement was performed according to the manufacturer's surgical protocol using the ADIN Dental Implant System (ADIN Dental Implant Systems Ltd., Afula, Israel). Osteotomy was performed sequentially using pilot and twist drills at a rotational speed of 500-1200 rpm under copious sterile saline irrigation. The osteotomy was extended approximately 2-3 mm beyond the root apex to obtain adequate primary stability while maintaining a prosthetically favorable implant angulation. Following insertion, the implant platform was positioned at the crestal level, and a cover screw was placed. The final insertion torque was recorded in Newton-centimeters at the time of implant placement for each implant.

PRF preparation and application

PRF was prepared immediately before implant placement, according to the original Choukroun protocol [5,11]. Approximately 10 mL of autologous venous blood was collected from the antecubital vein using sterile glass vacuum collection tubes without anticoagulants (BD Vacutainer®, Becton, Dickinson and Company, Franklin Lakes, New Jersey, USA). Immediately after collection, the tubes were transferred to a tabletop centrifuge (REMI Elektrotechnik Ltd., Mumbai, Maharashtra, India) and centrifuged at 3000 rpm for 10 min. Prompt centrifugation was performed to facilitate natural coagulation and fibrin polymerization.

After centrifugation, three distinct layers were obtained: red blood cells at the bottom, platelet-poor plasma at the top, and a PRF clot in between. The PRF clot was gently separated using sterile tissue forceps and scissors while preserving a thin erythrocyte layer attached to its base. Excess platelet-poor plasma was discarded, and the PRF clot was lightly compressed between sterile gauze to obtain a resilient fibrin membrane. The prepared PRF clot was placed in the residual gap between the implant surface and extraction socket walls (jumping gap), thereby occupying the peri-implant defect created during immediate implant placement. An additional PRF membrane was applied over the implant site before flap approximation was performed. Primary wound closure was achieved using interrupted sutures with 3-0 black silk sutures (Ethicon, Johnson & Johnson, Raritan, New Jersey, USA).

Postoperative management and follow-up

Standardized postoperative care was provided according to the institutional protocol, including prescribed antibiotics, analgesics, and oral hygiene instructions. Sutures were removed approximately 7-10 days after surgery. Archived follow-up records were reviewed at baseline, three months, and six months. Final prosthetic rehabilitation was performed after confirming satisfactory healing and osseointegration, according to the documented clinical protocol.

Assessment of implant stability

Implant stability was assessed using resonance frequency analysis (Osstell® Beacon, W&H, Göteborg, Sweden) and expressed as the Implant Stability Quotient (ISQ). The baseline ISQ represented primary implant stability and was obtained immediately after implant placement. Following a three-month submerged healing period, second-stage implant uncovering and prosthetic loading were performed, and the six-month ISQ was subsequently recorded three months after prosthetic loading. Thus, the six-month ISQ represented implant stability following osseointegration and an initial period of functional loading. The change in implant stability was calculated as the difference between the six-month and baseline ISQ values. A positive ΔISQ indicated an increase in implant stability from implant placement to the six-month assessment. When multiple measurements were available, the predefined mean of the valid readings was used for analysis.

Assessment of crestal bone level

Intraoral periapical radiographs obtained at implant placement, three months, and six months were retrospectively evaluated. Crestal bone level was measured as the vertical distance from the implant shoulder to the first radiographically identifiable bone-to-implant contact on the mesial and distal aspects of the implant. Implant-length calibration was used as an internal reference to reduce magnification-related measurement discrepancies. Because of the retrospective nature of the study, the archived records did not provide sufficient documentation to confirm the use of an individualized bite block, radiographic stent, or dedicated paralleling device for every radiographic examination. Therefore, projection geometry could not be assumed to have been identical across all time points. Mesial and distal measurements were recorded separately and used to calculate the overall crestal bone change. An increase in the distance between the implant shoulder and the first bone-to-implant contact was interpreted as crestal bone loss. The primary radiographic outcome was the total crestal bone change from baseline to six months. Additional interval changes were calculated from baseline to three months and from three to six months. An increase in the distance between the implant shoulder and the first bone-to-implant contact was interpreted as crestal bone loss. Radiographic assessments were performed using standardized imaging and measurement protocols documented in the archived clinical records.

Bone-density assessment

Preoperative CBCT records were used to determine the recipient-site bone density. Bone density was recorded in Hounsfield units (HU) using calibrated CBCT imaging software (BlueSkyPlan, version 4.0, Blue Sky Bio, Libertyville, IL, USA). A standardized region of interest corresponding to the planned implant recipient site was used to avoid cortical boundaries, adjacent tooth structures, metallic restorations, and other radiographic artifacts in the images. The continuous bone density value in HU was used as the principal radiographic predictor in the regression analysis, and the recipient sites were categorized as Type II or Type III according to the predefined eligibility criteria.

Examiner calibration and measurement reliability

All radiographic measurements were performed by a calibrated examiner (SKK) who was blinded to previous measurements and clinical outcome data. Twenty percent of the radiographic images were randomly selected and re-evaluated after a two-week interval under identical measurement conditions. Intra-examiner reliability was assessed using a two-way mixed-effects intraclass correlation coefficient with absolute agreement. The ICCs were 0.94, 0.92, and 0.95 at baseline, three months, and six months, respectively. A formal smallest detectable change could not be calculated because the raw paired repeated-measurement differences required to derive the standard error of measurement were not available in the retrospective records.

Study outcomes and predictor variables

The primary outcome was the total crestal bone change from implant placement to six months. Secondary outcomes included crestal bone change from baseline to three months, crestal bone change from three to six months, and change in ISQ from baseline to six months. Baseline variables considered as potential predictors of total crestal bone change at six months included age, sex, recipient-site bone density, final insertion torque, and baseline ISQ. Bone type was recorded descriptively, whereas continuous recipient-site bone-density measurements expressed in Hounsfield units (HU) were entered into the regression model. The six-month crestal bone assessment and ISQ measurement were performed after the three-month healing period and subsequent prosthetic loading, thereby reflecting early peri-implant remodeling and implant stability during the initial period of functional loading.

Statistical analysis

Statistical analyses were performed using SPSS Statistics for Windows, version 26.0 (IBM Corp., Armonk, New York, USA). Categorical variables were expressed as frequency and percentage. The normality of continuous variables was assessed using the Shapiro-Wilk test and summarized as mean ± standard deviation (SD). Changes in crestal bone levels between baseline and three months, three and six months, and baseline and six months were analyzed using paired-samples t-tests, as the distribution of paired differences satisfied the assumption of approximate normality. The change in ISQ from baseline to six months was evaluated using a paired-sample t-test. Mean differences, 95% confidence intervals, p-values, and Cohen's d effect sizes were reported.

Multiple linear regression analysis was performed to identify baseline factors independently associated with total crestal bone changes at six months. The total crestal bone change from baseline to six months was entered as the dependent variable, whereas the baseline ISQ, final insertion torque, bone density, age, and sex were entered as independent variables. Unstandardized regression coefficients, standard errors, standardized coefficients, 95% confidence intervals, t-values, p-values, coefficient of determination (R²), and adjusted R² were reported. The overall significance of the regression model was assessed using F-statistics.

The assumptions of linear regression were evaluated before interpretation, including linearity, independence of observations, homoscedasticity, normality of residuals, and multicollinearity. Variance inflation factor (VIF) values were examined to assess multicollinearity among the predictor variables. The regression analysis was considered exploratory because of the retrospective observational design and relatively modest sample size. Therefore, statistically significant associations were interpreted as associations rather than causal effects.

## Results

Study population and baseline characteristics

A total of 52 single-implants treated with adjunctive PRF were included in the final analysis. The mean age of the cohort was 48.6 ± 11.3 years, with 29 (55.8%) males and 23 (44.2%) females. Recipient sites were predominantly classified as type II bone, comprising 31 (59.6%), whereas 21 sites (40.4%) were classified as type III. The mean recipient site bone density was 612.4 ± 118.7 HU, the mean final insertion torque was 34.8 ± 6.9 Ncm, and the mean baseline implant stability quotient (ISQ) was 68.4 ± 4.2 (Table 1).

**Table 1:** Baseline demographic and clinical characteristics (n = 52) Continuous variables are presented as mean ± standard deviation (SD), whereas categorical variables are expressed as frequencies and percentages HU - Hounsfield units; ISQ - Implant Stability Quotient; Ncm - Newton‑centimeters

Baseline variable	Category	Value
Age (years)	Mean ± SD	48.6 ± 11.3
Sex	Male, n (%)	29 (55.8%)
	Female, n (%)	23 (44.2%)
Bone density (HU)	Mean ± SD	612.4 ± 118.7
Bone type	Type II, n (%)	31 (59.6%)
	Type III, n (%)	21 (40.4%)
Final insertion torque (Ncm)	Mean ± SD	34.8 ± 6.9
Baseline ISQ	Mean ± SD	68.4 ± 4.2

Crestal bone changes

Crestal bone changes were evaluated across predefined follow-up intervals using paired-sample t-tests. The mean crestal bone loss from baseline to three months was 0.18 ± 0.09 mm, with a statistically significant difference (t = 14.21, df = 51, p < 0.001; Cohen's d = 1.97). From three to six months, an additional mean crestal bone loss of 0.14 ± 0.08 mm was observed (t = 12.08, df = 51, p < 0.001; Cohen's d = 1.68). Overall, the mean crestal bone loss from baseline to 6 months was 0.32 ± 0.13 mm (t = 17.62, df = 51, p < 0.001; Cohen's d = 2.44), representing the primary radiographic outcome (Table 2).

**Table 2:** Change in crestal bone level across follow-up intervals (paired-samples analysis) *p < 0.05 was considered statistically significant. Positive mean change values indicate crestal bone loss (such as an increase in the implant‑shoulder‑to‑bone distance). Cohen's d was calculated as the mean difference divided by the SD of the differences. Paired‑samples t‑tests were employed, as the difference scores were approximately normally distributed. SD - standard deviation; df - degrees of freedom

Interval	Mean change ± SD (mm)	Test value	df	p-value	Effect size (Cohen's d)
Baseline → 3 months	0.18 ± 0.09	t = 14.21	51	< 0.001*	1.97
3 → 6 months	0.14 ± 0.08	t = 12.08	51	< 0.001*	1.68
Baseline → 6 months	0.32 ± 0.13	t = 17.62	51	< 0.001*	2.44

Change in implant stability

Implant stability increased significantly from baseline to six months. The mean ISQ increased from 68.4 ± 4.2 at implant placement to 74.1 ± 3.8 at six months. The mean increase in ISQ was 5.7 ± 3.1, with a 95% confidence interval of 4.8-6.6. This increase was statistically significant (t = 13.35, df = 51, p < 0.001, Cohen's d = 1.86) (Table 3).

**Table 3:** Change in implant stability quotient (ISQ) from baseline to six months *p < 0.05 was considered statistically significant. A paired-samples t-test was employed for within-group comparisons, as the distribution of the change scores satisfied the normality assumption. ΔISQ - change in Implant Stability Quotient (positive values denote increased secondary stability); SD - standard deviation; df - degrees of freedom; CI - confidence interval

Time point	Mean ISQ ± SD	ΔISQ, Mean ± SD (95% CI)	Test value (df)	p-value	Effect size (Cohen's d)
Baseline	68.4 ± 4.2	5.7 ± 3.1 (4.8–6.6)	t = 13.35 (51)	< 0.001*	1.86
6 months	74.1 ± 3.8				

Baseline predictors of crestal bone change

Multiple linear regression analysis was performed to identify baseline factors independently associated with total crestal bone changes at six months. The overall regression model was statistically significant and explained 44.6% of the variance in the total crestal bone change (R² = 0.446; adjusted R² = 0.386; F(5,46) = 7.42, p < 0.001). A higher baseline ISQ was significantly associated with lower crestal bone loss (β = -0.0110, 95% CI: -0.0190 to -0.0030; standardized β = -0.34; p = 0.006). Similarly, a higher final insertion torque was independently associated with lower crestal bone loss (β = -0.0060, 95% CI: -0.0100 to -0.0020; standardized β = -0.30; p = 0.015). Greater recipient site bone density was also significantly associated with lower crestal bone loss (β = -0.0003, 95% CI: -0.0005 to -0.0001; standardized β = -0.28; p = 0.022). In contrast, age (β = 0.0020, p = 0.240) and sex (β = 0.0140, p = 0.653) were not significant predictors. All variance inflation factor values were <2, indicating no multicollinearity among the predictors (Table 4).

**Table 4:** Multiple linear regression - baseline predictors of total crestal bone change at six months *p < 0.05 was considered statistically significant. All VIF values were < 2, confirming the absence of problematic multicollinearity. Negative β coefficients for baseline ISQ, insertion torque, and bone density indicate that higher baseline values were independently associated with less crestal bone loss at the six‑month follow‑up. SE - standard error; VIF - variance inflation factor; CI - confidence interval; HU - Hounsfield units; ISQ - Implant Stability Quotient; Ncm - Newton-centimeters

Predictor variable	β (unstandardized)	SE	β (standardized)	95% CI	t value	p-value	VIF
Baseline ISQ	-0.0112	0.0039	-0.34	-0.0190 to -0.0034	-2.86	0.006*	1.21
Insertion torque (Ncm)	-0.0051	0.0020	-0.30	-0.0092 to -0.0011	-2.53	0.015*	1.18
Bone density (HU)	-0.00024	0.0001	-0.28	-0.00045 to -0.00004	-2.37	0.022*	1.34
Age (years)	0.0019	0.0016	0.14	-0.0018 to 0.0056	1.19	0.240	1.15
Sex (male vs. female)	0.0136	0.0305	0.05	-0.0478 to 0.0759	0.45	0.653	1.09

Intraexaminer reliability

The intraexaminer reliability for crestal bone measurements was excellent at all assessment time points. The ICC was 0.94 (95% CI: 0.89-0.97) at baseline, 0.92 (95% CI: 0.86-0.96) at three months, and 0.95 (95% CI: 0.90-0.98) at six months. All reliability estimates were statistically significant (p < 0.001), supporting the high reproducibility of the radiographic measurements (Table 5).

**Table 5:** Intraexaminer reliability of crestal bone measurements *p < 0.05 was considered statistically significant, the intraclass correlation coefficient (ICC) was calculated on a calibration subset comprising approximately 20% of the images, which were re‑measured after a masked interval in accordance with the blinding and reliability protocol, an ICC value ≥ 0.90 was conventionally interpreted as indicating excellent measurement reproducibility.

Assessment time point	ICC (two-way mixed, absolute agreement)	95% CI	Test value (df)	p-value
Baseline	0.94	0.89–0.97	32.4 (10,10)	< 0.001*
3 months	0.92	0.86–0.96	25.7 (10,10)	< 0.001*
6 months	0.95	0.90–0.98	38.1 (10,10)	< 0.001*

## Discussion

The present retrospective observational cohort study evaluated crestal bone changes and implant stability over a six-month period among immediate implants treated with adjunctive PRF, and further examined baseline clinical and radiographic factors associated with crestal bone change. Overall, the findings indicated limited early crestal bone remodeling, accompanied by improved implant stability during the healing period. More importantly, greater primary implant stability, higher insertion torque, and greater recipient site bone density were associated with better crestal bone maintenance, whereas demographic characteristics were not significantly associated. Multivariable analysis further indicated that the combination of these baseline mechanical and radiographic characteristics accounted for a substantial proportion of the variability in early crestal bone changes. These findings suggest that the local osseous environment and mechanical conditions established during implant placement may be more relevant to early crestal bone maintenance and implant success than demographic factors [12].

Crestal bone remodeling following immediate implant placement is clinically important because preservation of the peri-implant marginal bone contributes to the maintenance of implant stability, soft tissue architecture, and long-term esthetic outcomes [3,4]. Immediate implant placement does not prevent physiological remodeling of the extraction socket, and early dimensional changes may occur during the transition from extraction socket healing to peri-implant osseous integration [13]. In the present cohort, the total mean crestal bone loss (0.32 mm over six months) indicated a relatively limited magnitude of early marginal bone change. The observed change was progressive across both follow-up intervals, with a greater absolute change occurring during the first three months. This temporal pattern is consistent with the concept that the early post-extraction period represents an active phase of socket remodeling and peri-implant tissue adaptation [14].

The use of PRF in the present cohort provides an important biological context for interpreting these findings. PRF is a second-generation platelet concentrate consisting of a fibrin matrix containing platelets, leukocytes, cytokines, and growth factors. The gradual release of biologically active mediators from the fibrin matrix may support angiogenesis, fibroblast proliferation, collagen synthesis, and osteoblastic activity [5,6]. In immediate implant placement, the PRF clot can occupy the residual jumping gap between the implant surface and socket wall and provide a biological matrix during the early healing phase [15,16]. Previous clinical investigations have reported favorable marginal bone preservation and peri-implant healing following immediate implant placement with PRF [17-20]. A systematic review also reported evidence supporting the potential role of PRF in alveolar ridge preservation and early osseointegration, although the overall evidence was heterogeneous [10]. These biological and clinical observations provide a plausible context for the favorable early bone changes observed in the present cohort.

However, the present findings should not be interpreted as evidence that PRF itself caused the observed preservation of crestal bone. PRF was routinely incorporated into the immediate implant protocol in a clinical setting, and all implants in the present cohort received PRF. As there was no non-PRF comparison group, the additional effect of PRF could not be isolated from the effects of immediate implant placement, surgical technique, primary stability, bone quality, and other aspects of clinical management. Thus, the principal contribution of the present study is not the assessment of the comparative efficacy of PRF but rather the identification of baseline factors associated with crestal bone maintenance among immediate implants treated with PRF.

An important finding was the significant increase in implant stability during the six-month period. The mean ISQ increased by 5.7 points from baseline to six months, indicating improved mechanical stability during the healing and osseointegration periods. A similar finding was reported in a previous study, where placement of immediate implants with PRF led to an increase in implant stability and bone density [21]. This increase is biologically plausible because primary implant stability is initially determined predominantly by mechanical engagement between the implant and surrounding bone, whereas secondary stability develops progressively through bone healing and remodeling [22]. Therefore, the observed increase reflects a transition from a mechanically dependent primary stability to a biologically mediated secondary stability. Importantly, the baseline ISQ was also independently associated with subsequent crestal bone changes in the regression model, suggesting that the initial mechanical environment of the implant may have implications for early marginal bone remodeling.

A higher baseline ISQ was associated with lower total crestal bone change at six months. Higher baseline ISQ reflects greater primary implant stability and may indicate more favorable mechanical engagement between the implant and the recipient bone [23]. Greater stability may reduce excessive micromotion during the early healing period, thereby providing a more favorable environment for bone-to-implant contact and osseointegration. However, although this association was statistically significant, its clinical magnitude should be interpreted cautiously because the observed effect was small relative to the precision limitations of the radiographic measurement method. The present study did not establish an absolute radiographic measurement error or minimum detectable change; therefore, the clinical importance of this association cannot be conclusively determined. Furthermore, because the study was retrospective and observational, this association should not be interpreted as demonstrating that increased ISQ directly prevents crestal bone loss.

The final insertion torque was also a significant independent predictor. A higher insertion torque was associated with lower crestal bone loss at six months. The insertion torque is an intraoperative measure of resistance encountered during implant placement and is commonly considered an indicator of primary mechanical stability [24]. Therefore, the relationship observed in the present cohort is consistent with the findings for the baseline ISQ, as both parameters provide complementary information regarding the mechanical stability of the implant at placement. Sarfaraz et al. [25] reported a significant positive correlation between the insertion torque and ISQ, with higher insertion torque values associated with greater implant stability. Their findings also demonstrated that baseline insertion torque was significantly related to subsequent ISQ measurements during healing. This association suggests that implants achieving greater mechanical stability at the time of insertion may experience more favorable early crestal bone maintenance in the implant group. However, insertion torque should not be considered an isolated determinant of treatment outcome because an excessively high insertion torque may also reflect excessive compression of surrounding bone. The present observational data support an association within the range of torque values represented in this cohort rather than a specific optimal torque threshold.

Recipient-site bone density was statistically associated with crestal bone change at six months, with higher bone density associated with lower crestal bone loss. This finding is consistent with the potential mechanical contribution of the local bone environment to implant stability, as greater bone density may provide greater resistance to implant micromotion and mechanical support during the early healing phase [26]. However, the magnitude of the observed association was very small and should not be interpreted as evidence of a clinically meaningful effect. Given the precision limitations of the radiographic measurement method, the estimated difference associated with variation in bone density may be below the level of change that can be reliably resolved radiographically. Although continuous bone density measurements in Hounsfield units were used rather than relying solely on categorical bone classification, this approach may have captured statistical variation in the regression model without necessarily indicating a clinically detectable difference in crestal bone maintenance. Therefore, the association should be interpreted as a statistical finding rather than as a clinically useful predictor of crestal bone change.

Clinical implications

Preoperative assessment of bone density and intraoperative evaluation of insertion torque and ISQ may provide supplementary information regarding primary implant stability and early crestal bone changes in immediate implant cases treated with PRF. However, given the small magnitude of the observed associations, these parameters should not be considered sufficient to guide decisions regarding implant placement, surgical planning, or prosthetic loading timing on the basis of the present findings alone. Their potential clinical utility requires further evaluation in prospective studies with standardized measurement methods and clinically meaningful outcome thresholds.

Limitations

The present findings should be interpreted in light of several limitations. The retrospective, single-center design and relatively small sample size limit causal inference and generalizability, whereas the six-month follow-up primarily reflects early peri-implant remodeling rather than long-term outcomes. Although clinically relevant predictors were included, other factors, such as implant position, socket morphology, implant dimensions, soft tissue phenotype, and prosthetic loading, could not be fully accounted for. Furthermore, bone density was estimated from the CBCT-derived grayscale values expressed as HU-like measurements. Unlike conventional CT, CBCT does not provide standardized and directly comparable HU values, and these measurements may vary with the scanner, acquisition parameters, field of view, artifacts, and software calibration [27]. Therefore, the bone density values in the present study should be interpreted as relative radiographic estimates, rather than absolute bone density measurements. Conventional CT with standardized HU calibration provides a reliable reference for quantitative assessment of bone density. Selection bias cannot be excluded because the primary analysis was restricted to implants with an available six-month crestal bone assessment, while implant failures and cases lost to follow-up were not included in the outcome analysis. Consequently, the reported estimates of crestal bone maintenance may be influenced by differential availability of follow-up data and should not be interpreted as representing the outcomes of all initially eligible implants.

Radiographic measurement limitations should also be considered. Although repeated measurements of a subset of radiographs demonstrated high intra-examiner reliability, this assessment primarily reflects measurement or reading error and does not quantify reproducibility of radiographic acquisition, projection geometry, or patient positioning between different time points. This limitation is particularly relevant when interpreting the relatively small longitudinal changes in crestal bone level (0.14-0.18 mm). Furthermore, because all radiographic measurements were performed by a single examiner, inter-examiner reliability could not be assessed.

Strengths

Despite these limitations, this study had several strengths. This study evaluated a relatively homogeneous cohort of immediate implants treated according to a consistent PRF protocol and surgical approach, used objective measures of implant stability and bone density, and incorporated multivariate analysis to identify independent baseline associations with crestal bone changes. These findings suggest that assessment of the recipient-site bone environment and achievement of adequate primary implant stability may be important considerations when anticipating early peri-implant crestal bone remodeling. The results also emphasize that the biological adjunct alone should not be considered independent of the mechanical and local osseous conditions present during implant placement.

Future recommendations

Future studies should prospectively evaluate larger and more diverse populations and incorporate appropriate comparison groups, including immediate implants without PRF and alternative regenerative materials, where clinically appropriate. A longer follow-up period is also required to determine whether the associations between baseline ISQ, insertion torque, bone density, and early crestal bone change persist over the functional life of the implant. Standardized assessments of socket morphology, facial bone thickness, implant position, soft tissue phenotype, and prosthetic loading would further improve the adjustment for potential confounding factors. Such studies would help determine whether the baseline predictors identified in the present cohort have consistent prognostic value across different clinical settings.

## Conclusions

Within the limitations of this retrospective observational cohort study, immediate implant placement with adjunctive PRF was associated with limited early crestal bone loss and improved implant stability over a six-month follow-up period. A higher baseline ISQ, greater insertion torque, and higher recipient site bone density were associated with better crestal bone maintenance. However, because PRF was routinely used in all cases and no non-PRF control group was available, the independent effect of PRF could not be established. Larger controlled prospective studies with longer follow-up periods are warranted to validate these findings and determine the comparative clinical effectiveness of PRF in immediate implant therapy.
